# Clarity and consistency in government-funded implementation strategies associated with greater evidence-based practice reach: a mixed-method comparative case study

**DOI:** 10.1186/s13012-025-01470-3

**Published:** 2025-12-22

**Authors:** Matthew Lee, Sarah B. Hunter, Baji Tumendemberel, Mekdes Shiferaw, Mark D. Godley, Jonathan Purtle, Gregory A. Aarons, Alex R. Dopp

**Affiliations:** 1 Department of Population Health, New York University Grossman School of Medicine, 180 Madison Avenue, New York, NY 10016 USA; 2 RAND, 1776 Main Street, Santa Monica, CA 90401 USA; 3 Chestnut Health Systems, 448 Wylie Drive, Normal, IL 61761 USA; 4 Department of Public Health Policy & Management, New York University School of Global Public Health, 708 Broadway, New York, NY 10003 USA; 5 Department of Psychiatry and Altman Clinical and Translational Research Institute Dissemination and Implementation Science Center, University of California San Diego, 9500 Gilman Drive (0812), La Jolla, San Diego, CA 92093 USA

**Keywords:** Youth substance use, Substance use disorder treatment, Evidence-based practice, A-CRA, Implementation, Reach, Policy, Case study methods, Behavioral health service systems, Financing strategies

## Abstract

**Background:**

Policymakers need research-informed guidance on leveraging national government funding to promote evidence-based practice (EBP) implementation, however empirical studies of policy financing strategies in implementation science remain limited. Major investments are already being made. Starting in 2012, the U.S. Substance Abuse and Mental Health Services Administration (SAMHSA) funded state substance use service agencies to implement EBPs for youth substance use. We examined 19 states funded to implement the Adolescent Community Reinforcement Approach (A-CRA), an exemplar EBP selected by most states. Using the Exploration, Preparation, Implementation, Sustainment (EPIS) Framework, we sought to explain state-level variation in A-CRA reach (defined as the proportion of A-CRA certified providers) and to identify policy implications for improving EBP financing strategies.

**Methods:**

We conducted an explanatory sequential mixed-method (QUAN→QUAL) comparative case study, treating each state as a case. States were categorized as achieving high, medium, and low reach during their grant periods using A-CRA certification records and state demographic data. We then synthesized available data (i.e., interviews with 33 state agency administrators, grant administrative records, other documents describing A-CRA implementation) to summarize grant activities completed and their quality, and factors potentially influencing reach in each state. Finally, we compared and contrasted state cases to identify policy implications through pattern matching techniques.

**Results:**

We characterized the 19 states’ reach levels as high (*n* = 7), medium (*n* = 5), and low (*n* = 7) and identified an average of 5 grant-related activities completed per state; the most common being A-CRA training to treatment organizations. Six states were case anomalies (e.g., low quantity and quality of activities, while achieving high reach). Most notably, we found that high-reach states had more specific, intentional, and explicit A-CRA implementation requirements for treatment organizations than did low- and medium-reach states. States were also more successful in achieving A-CRA reach when they reported proactively addressing implementation barriers (e.g., provider turnover, state leadership buy-in and support).

**Conclusions:**

Our mixed-method comparative case study advances policy-focused implementation research related to EBP financing strategies, demonstrating how examination of large-scale real-world funding initiatives can produce generalizable lessons. Our findings have implications for how future funding initiatives can facilitate EBP delivery to maximize reach.

**Supplementary Information:**

The online version contains supplementary material available at 10.1186/s13012-025-01470-3.

Contributions to the literature

- By examining patterns within and across reach levels in 19 states, we generated explanations highlighting the importance of policymakers and funders being specific, intentional, and explicit in their expectations and deliverables – which enabled some states to address widely recognized implementation barriers like staff turnover.
- Our work advances understanding of how to promote reach of evidence-based practices for youth with substance use problems, for whom access to effective treatment remains low.
- This study demonstrates how comparative case study methods can add value to policy implementation research by richly characterizing outcomes (high, medium, and low reach) and processes (grant activities).

## Background

National governmental agencies in the United States (U.S.) and other countries invest in evidence-based practice (EBP) implementation through financing strategies, such as grants and contracts, that provide resources to service delivery organizations, health systems, and their partners [1–3]. However, there is a lack of research-informed guidance on how to leverage financing strategies to optimize implementation [4] across priority health and social services – including youth substance use treatment, a service sector in which access to effective treatment remains low [5–8]. Further, while there have been recent advances in policy-focused implementation science [9–16], empirical work, such as examining the role of financing as a policy implementation strategy, remains limited [17–20].

To date, one of the largest investments in widespread EBP implementation for youth substance use disorder (SUD) treatment has been through the U.S. Substance Abuse and Mental Health Services Administration (SAMHSA). An exemplar EBP for treating youth SUD, and one of the most commonly selected EBP options by SAMHSA grantees, is the Adolescent Community Reinforcement Approach (A-CRA) [21], a 12–14 week behavioral treatment for 12–25 year olds. A-CRA uses common EBP components for youth SUD, including: replacing environmental factors that support substance using behaviors with client-selected prosocial activities and behaviors, opportunities for delivery in community settings, and procedures to address family and community influences. Several randomized trials have shown A-CRA reduces substance use and improves mental health and social outcomes [22–27]. A-CRA has been widely implemented in the U.S. where it was developed, as well as in Brazil, Canada, Iran, the Netherlands, and Spain [28–30].

Over time, SAMHSA issued two types of grant funding that supported A-CRA implementation: first, “organization-focused” grants that directly funded treatment organizations, starting in 2006; and later, starting in 2012, “state-focused” grants that provided funding to state substance use agencies to develop state infrastructure with the intent of better facilitating EBP implementation (in partnership with local treatment organizations). The infrastructure elements required for state-focused grants varied by funding announcement, but generally included hiring a full-time project manager, providing EBP training, developing a financial map or other fiscal planning, collaborating across state youth-serving agencies, engaging youth and families, and policy and regulation development to support EBP implementation. Prior research has shown that state-focused grants achieved lower levels of provider-level reach – defined as the proportion of trained providers (clinician or clinical supervisor working in a treatment organization) who received A-CRA clinical delivery and/or supervision certification – compared to organization-focused grants [31]. However, there was notable variation in reach rates by state, and federal and state officials who reviewed our findings expressed interest in drawing on successes from state-focused grants to inform future funding initiatives [32].

The present study used comparative case study methods [33] to comprehensively examine state infrastructure and A-CRA implementation efforts through the SAMHSA grants. Other applications of these methods have evaluated EBP implementation and sustainment [17, 34, 35], as well as policy- and systems-level efforts to implement and sustain change [36–39]. We focused on reach as the primary implementation outcome, as it represented the extent to which each grant-funded state agency was able to meet adolescent substance use treatment needs in their state. Achieving high reach would require states to move beyond prevailing approaches that consist of sending staff to training or using asynchronous limited training, akin to “train and hope” approaches where there is an unfounded expectation that attending training is sufficient to build knowledge and skills that lead to high-fidelity EBP implementation and sustainment [40]. By treating each state as a case, a comparative case study approach allows for deriving patterns within and across states to achieve understanding of what contributed to A-CRA reach variation.

## Methods

Data were drawn from our study comparing A-CRA implementation and sustainment outcomes of state-focused versus organization-focused grants (see published study protocol [41]). Herein we describe details relevant for the comparative case study analysis, which focused on the state-focused grantees (also see Standards for Reporting Implementation Studies [42]; Additional file 1). All procedures were approved by the RAND IRB (Protocol #2020-N0887).

### Study overview

We used an explanatory sequential mixed-method (QUAN→QUAL) comparative case study design [43]. Our research questions were: (1) How did states use their SAMHSA grant funding to support A-CRA implementation?; (2) Does variation in grant-funded activities help explain state-level variations in A-CRA reach?; and (3) Do contextual differences within and across the states help to explain reach outcomes?

### Project context

This study is grounded in the Exploration, Preparation, Implementation, Sustainment (EPIS) framework [44, 45], which operationalizes four phases by which settings (e.g., state substance use agencies and treatment organization sites within their jurisdiction) implement EBPs such as A-CRA. The EPIS framework provides four constructs to understand the implementation process: 1) *Outer Context* (extra-organizational factors such as service and policy environment characteristics, as well as characteristics of the EBP recipients – i.e. youth receiving A-CRA treatment); 2) *Inner Context* (intra-organizational factors such as leadership, resources, staffing, and characteristics of the EBP deliverers – i.e. providers who are trained, certified, and then deliver A-CRA treatment); 3) *Bridging Factors* at the interplay between the Outer and Inner Context (e.g., the SAMHSA state-focused grant requirements + funding, partnerships, inter-connections, and characteristics of state-level intermediaries, contracting); and 4) *Innovation Factors* of the EBP – i.e. A-CRA characteristics.

We operationalized EPIS in two ways: 1) to retrospectively categorize SAMHSA’s decision-making process in a structured way, and 2) to guide our comparative case study design focused on how grant-funded state substance use agencies used their SAMHSA funding. We consider the Exploration phase of each state-focused grant initiative to be when SAMHSA selected EBPs, including A-CRA, to be the focus for a given funding opportunity and states applied for that funding. We consider the Preparation phase to begin when states received the grants, up to when active EBP implementation began. We included grantees from three state-focused grant initiatives issued between 2012–2017: the State Adolescent Treatment and Dissemination Initiative (SAT-ED), the State Adolescent and Transitional Youth Treatment and Dissemination Initiative (SYT-I), and the State Youth Treatment Initiative (SYT). We also included one grantee from a different (2-year) SAMHSA grant initiative (i.e., Opioid State Targeted Response effort) that used the support to train providers in their state in A-CRA.

We consider the Implementation phase to be A-CRA implementation during the active grant funding period. The grant mechanism was designed to: 1) train and certify A-CRA clinicians and supervisors at treatment organizations throughout a state (*inner context* focus on intra-organizational factors); and 2) develop state infrastructure to facilitate A-CRA delivery (*outer context* focus on extra-organizational factors and *bridging factors* that connect the SAMHSA-funded state agencies and participating treatment organization sites). During this phase, Chestnut Health Systems (CHS) provided A-CRA training and certification for clinicians and supervisors [46]. This study focused on the Implementation phase as our Reach outcomes reflect certifications achieved during each state’s grant period.

### Data sources and collection procedures

Primary and secondary data sources were: semi-structured interviews conducted with state agency administrators (primary), CHS certification records (secondary), and documents related to state substance use funding, grant- or ACRA-related activities in the state (secondary).

#### Semi-structured interviews

Trained research staff conducted interviews between November 2021-June 2022 with administrative staff from the grant-funded state agencies. Group interviews were conducted when multiple administrators from the same state participated. The interview guide consisted of open-ended questions and standardized probes (see Additional file 2). Interviewers asked about the supports and infrastructure for A-CRA implementation established during the funding period based on the specifications detailed in the grant announcement, and contextual influences on A-CRA implementation success. Interviews lasted approximately 45–60 min and were audio-recorded; we then created verbatim, de-identified transcripts. Participants were offered a $25 USD incentive for each interview, although some participants declined the incentive. The final sample included 33 state administrators across 19 states.

#### Certification records

CHS maintains a database of A-CRA training and certification completion dates. From these records, we were able to calculate how many providers (clinician or clinical supervisor) from each treatment organization and state achieved an A-CRA certification during the grant period. Completion of the certification process required not only formal adoption, but also demonstration of adequate fidelity to the A-CRA model.

#### State document review

During the interviews, we asked participants to share documents that provided details on state grant-funded activities. The lead researcher (ARD) also conducted supplemental web searches to identify relevant documents on state agency websites. Examples of documents collected included annual behavioral health reports, grant progress and evaluation reports. We were not able to systematically obtain the same document types for every state. A total of 141 documents were included, with no documents sourced for three states. The average number of documents collected across the other 16 states was 8.81 (range: 1–30).

### Comparative case study approach

We considered each of the 19 states as a case, as this is the natural unit of study for grant recipients. For each state case, we synthesized available data into a descriptive summary using a case summary template (see Additional file 3). This followed an explanatory sequential mixed-method design (QUAN→QUAL) with the qualitative sources of data (interviews, documents) being used to build explanations for the initial quantitatively-derived reach outcome calculations, where explanation building involved identifying patterns that could explain reach levels [47].

#### Reach outcomes and classification + state characteristics

The first portion of the template documented the state’s reach level, as well as state-level characteristics during the grant period: youth SUD prevalence, annual substance use services budget (total and per capita spending), and Medicaid Expansion status (see Additional file 4 for data sources).

Reach level was the basis for our pattern matching approach. We operationalized reach by calculating the proportion of treatment organizations, out of all those trained during a state’s grant period, where at least one provider obtained any A-CRA certification by the end of grant funding. Furthermore, we considered the number of youth with SUD that certified A-CRA providers would need to treat to provide statewide coverage. Specifically, we calculated the ratio of the number of certified providers (i.e., the numerator of the reach rate) and estimates of youth with SUD from the first year of the active grant period using SAMHSA National Survey on Drug Use and Health (NSDUH) [48] which provides U.S. data on SUD prevalence. Using these calculations, we defined three levels of reach – *high*,* medium*, and* low* – as specified in Table 1. A state had to meet the specified thresholds for certification rate and ratio to youth with SUD to be designated as having medium or high (as opposed to low) reach.

**Table 1:** Reach categorization criteria

Reach Level	Certification Rate	Rate of Youth w/ SUD per 1 Certified A-CRA Provider
High	≥ 50% of organizations trained had at least 1 certified provider AND	< 500 youth
Medium	30–49% of organizations trained had at least 1 certified provider AND	< 2000 youth
Low	< 30% of organizations trained had at least 1 certified provider OR	> 2000 youth

#### Case summary completion

The remaining sections of the template summarized information from the interviews and documentation. For states with limited information (e.g., interviewed administrative staff not employed during grant period, no documentation available), we specifically sought insight from CHS.

Our template captured whether and to what extent each state conducted nine activities specified in the grant funding announcements, detailed in Table 2. Activities were assigned a “1” if evidence indicated that they were completed and a “0” if not completed. In some instances, partial credit (“0.5”) was assigned, for example if a full-time staff person was hired for a portion of, but not the full, grant period. Activities that could not be confirmed by any data source were labelled “M” for “missing.”

**Table 2:** Grant-funded activities specified in the state-focused grant mechanism

Element/Activity Name	Case Template Questions
1. Full-time staff position	• Who was involved? • Did the state hire at least 1 full-time staff position dedicated to managing this program? • Was there turnover during the grant period?
2. Training requirements	• Did the state provide A-CRA training to designated treatment organizations? • Did they create a state-wide multi-year workforce training implementation plan? • Did they provide cross-agency training? • Did they provide continuing education events?
3. Policy and regulation development	• Did the state develop policies or regulations to promote the use of A-CRA?
4. Site sustainability planning	• Did the state support treatment organizations’ sustainability planning for the use of A-CRA?
5. State-specific strategic financial planning/financial mapping	• Did the state conduct strategic financial planning (i.e. conduct financial mapping) to support funding sources/payment for A-CRA implementation?
6. Youth/Family engagement structures	• Did the state support treatment organizations’ efforts to promote youth/family engagement? • Did they include youth and families in their coordination efforts?
7. Inter-organizational collaboration	• Did the state promote treatment organizations’ capacity for collaboration?
8. Computer systems/Electronic Health Record (EHR) improvement	• Did the state promote treatment organization in making improvements to their computer systems or EHRs?
9. Other activities	• Were there any other activities that the state agency provided to support A-CRA implementation?

The SAMHSA funding opportunity numbers were: TI-12–006, TI-13–014, TI-15–004, TI-17–002

The template also captured which contextual factors across EPIS constructs (*Outer Context*, *Inner Context*, *Bridging Factors*, *Innovation Factors*) were most related to reach; the extent the participants were satisfied with A-CRA reach; and information on three additional implementation outcomes: *Grant Fidelity* (factors that impacted adherence to funding requirements)*, Adoption* (how the state selected implementing treatment organizations), and *Fit* (related to youth, family and/or community acceptability, feasibility, and appropriateness).

The template also captured whether the reach rate observed was a match or mismatch with the total *quantity* (number and percentage) of grant-related activities completed, and their *quality* (extent to which the activity was not only completed, but also with a robust effort to guide or improve implementation). An example of higher quality was multiple rounds of financial mapping, including multiple state actors in the process, to inform ongoing decision-making. By contrast, an example of a lower quality activity was conducting a single round of financial mapping that was not used to guide implementation. We were able to assess quality when the participants had managed the grant for the full period and could describe both failed and successful attempts at completing activities, as well as when state documents captured relevant details. For a small number of cases (States 1, 2, 6, 19), quality was difficult to assess due to not being able to interview all eligible administrators who managed the grant *and* having few to no state documents.

Two experienced researchers (ML, SBH) completed a case summary together to pilot the template, with guidance and supervision from the PI (ARD). After making template refinements, they then independently completed a subset of the remaining 18 cases. Each case summary was reviewed by at least one additional team member (ARD, BT) for completeness and clarity. CHS collaborators (led by MDG) who had actively supported states and trained providers during their active grant period helped to verify and validate elements for case descriptions as needed, as well as provide additional context.

#### Pattern matching

Following completion of all case summaries, we compared and contrasted across the 19 states to *pattern match* for implementation processes and outcomes [33]. We examined the low-reach state cases together, then did the same for the medium-reach and high-reach states. Finally, we examined patterns across reach levels. Based on the match/mismatch analysis from the case summaries, we also identified case “anomalies” [33], defined as state cases where quantity and quality of grant-funded activities did not match the reach level (e.g., a low-reach state completing many activities with high quality). We identified anomalies within all three reach levels, so simple pattern matching was insufficient. Our goal was to develop the most complete explanations possible that held across all levels of reach and all 19 state cases – anomalies included – so we extended into a special type of *pattern matching* called *explanation building* [33, 47] that seeks to develop rich explanations that account for all case observations. We prioritized explanations that best represented all available evidence and the most significant aspects of state experiences, beyond just counting activities, which allowed us to consider and rule out plausible or rival explanations for those same observations. Incorporation of case anomalies into our explanations allowed for even more robust clarification of factors that affected successful implementation.

## Results

Across the 19 cases, 7 were classified as low reach, 5 as medium reach, and 7 as high reach based on the criteria outlined in Table 1. The grant period length ranged from 2 to 6 years (mean = 4.32 years), with 7 states having two rounds of funding, and 12 states only having one. Organized by reach level, Table 3 displays the number of grants and total length of grant period for all 19 states, as well as summarizes state characteristics (youth SUD prevalence, state substance use budget, Medicaid expansion status). None of the characteristics had a pattern that helped explain state reach levels.

**Table 3:** State characteristics by reach level

State ID	Reach Level	# of Grants	# of Grant Years	Youth SUD Prevalence *(Grant Start Year)*	Youth SUD Prevalence *(Grant End Year)*	State Substance Use Budget** *(USD)*	State Substance Use Budget*** *(Per Capita)*	Medicaid Expansion Status *(during grant)*
1	Low	1	3	6.05%	2.69%	$16,405,000	High	Y
2	Low	1	3	6.44%	4.00%	$21,524,000	Low	Y
7	Low	2	6	4.79%	3.47%	$86,027,000	Med	Y
11	Low	1	3	5.61%	2.58%	$117,030,000	High	Y
13	Low	1	5	2.79%	8.53%	$31,271,000	Low	Y
18	Low	1	4	2.29%	5.07%	$10,221,000	Low	N
19	Low	1	4	2.53%	6.61%	$47,629,000	High	N
4	Med	1	3	5.63%	4.22%	$24,954,000	Low	Y
6	Med	1	3	5.59%	2.82%	$18,633,000	Med	N
9	Med	2	6	6.07%	2.45%	$35,750,000	Med	Y
16	Med	2	6	5.74%	2.96%	$6,439,000	Low	N
17	Med	1	3	4.67%	2.96%	$25,469,000	Low	N
3	High	1	4	2.83%	8.7%	$185,182,000	High	Y
5	High	1	2*	3.32%	3.6%	$14,848,000	Low	Y
8	High	2	6	5.12%	3.31%	$14,309,000	Low	Y
10	High	2	6	5.55%	3.30%	$90,107,000	Med	Y
12	High	2	6	5.20%	3.80%	$14,072,000	Low	Y
14	High	1	3	4.93%	2.53%	*Data not reported*	*Data not reported*	N
15	High	2	6	4.87%	3.18%	$36,246,000	Med	N

In some cases, the grant award period includes a no-cost extension (NCE) period

^*^ State 5 used a different grant mechanism that was only 2-years to support A-CRA implementation

^**^ State substance use budgets are from 2015, which was an active grant year for 15 of the 19 states

^***^ State substance use spending per capita was categorized as high if within the top one-third percentile (tertile), medium if in the second tertile, and low if in the bottom tertile

Table 4 summarizes grant-funded activities by state and reach level. The most frequently completed activities were training to treatment organizations (*n* = 18), hiring a full-time grant manager (*n* = 14.5), and promoting inter-organizational collaboration (*n* = 13). The least completed activities were policy and regulation development (*n* = 9), site sustainability planning (*n* = 9), computer systems/EHR improvements (*n* = 7), and other activities (*n* = 5). Average number of completed activities was 5.03 (range: 1.5–9) and average completion percentage was 57.5% (range: 16.7–100%).

**Table 4:** Overview of state cases by reach level and grant-funded activities completed

State ID	1	2	7	11	13	18	19	4	6	9	16	17	3	5*	8	10	12	14	15
Reach Level	Low	Low	Low	Low	Low	Low	Low	Med	Med	Med	Med	Med	High	High	High	High	High	High	High
Grant-funded Activity																			
Training requirements	1	1	1	1	0	1	1	1	1	1	1	1	1	1	1	1	1	1	1
Full-time staff position	M	1	1	1	1	1	1	M	M	0.5	1	1	0.5	0.5	1	1	1	1	1
Inter-organization collaboration	0	1	0	1	1	1	0	0	1	1	1	0	1	0	1	1	1	1	1
Strategic financial planning	0	1	0	0	1	M	M	0	0	1	1	1	1	0	1	1	1	1	M
Family/youth engagement	1	1	0	1	1	0	0	1	0	0	1	0	1	0	0	1	1	1	0
Policy + regulation development	0	1	0	0	1	0	1	1	0	0	1	0	1	0	1	1	0	0	1
Site sustainability planning	1	1	0	1	1	0	1	0	0	0	1	0	1	0	0	1	0	0	1
Computer systems/EHR improvement	0	1	1	0	0	0	1	0	0	0	1	1	1	0	1	0	0	0	1
Other activities	1	0	0	0	0	0	0	0	0	0	1	0	1	0	1	0	0	1	0
Completion (%)**	50.0	88.9	22.2	55.6	66.7	37.5	62.5	37.5	25.0	38.9	100	44.4	94.4	16.7	77.8	77.8	55.6	66.7	75.0

1 = full credit for completing the activity

0.5 = partial credit for completing the activity

0 = no credit for the activity

M = “missing”/unable to assign credit for the activity

^*^ State 5 used a different grant mechanism that was 2-years to support A-CRA implementation. Criteria + standards were different

^**^States with a missing element did not have that factored into the denominator when calculating the completion percentage

Notably, States #1 (low reach), 4 (medium reach), and 11 (low reach) all opted to establish sub-contracts with an external organization in the state to administer grant activities. Such organizations included regional managed health care organizations, an intermediary organization that supports EBP implementation, and a university-based training and research center.

### Case anomalies

We identified 6 total case anomalies: 3 low reach, 2 medium reach, and 1 high reach. Table 5 provides the average percentage of activities completed by reach level for all states, and with anomaly states excluded.

**Table 5:** Average activities by reach level

*Low reach* – Average percentage of activities completed for all states: 54.8%
Average with anomaly states excluded: 43.1%
*Medium reach* – Average percentage of activities completed for all states: 49.2%
Average with anomaly states excluded: 60.6%
*High reach* – Average percentage of activities completed for all states: 66.3%
Average with anomaly states excluded: 74.6%

One of the seven high-reach states (State 5) was a case anomaly meaning that they completed a lower quantity of grant activities (16.7%) but still achieved high reach. However, this could be explained by them obtaining funding through a different mechanism that they used to support for provider training and certification without requiring the pursuit of other activities.

Medium-reach anomalies were more difficult to assess, as determining moderate quantity or quality of activities was sometimes less clear than assessing high and low. Ultimately, we identified two out of five medium-reach states (States 9 and 16) as case anomalies with activities that did not match their attained reach. State 16 completed all 9 activities yet only attained medium reach, likely due to issues related to provider shortages and turnover within their state that impacted their final reach rate. State 9 had a lower quantity of activities (38.9%) that were also considered lower quality, with the state attempting to do many activities but acknowledging that they were not successful in completing many of them. Note that State 6 had the lowest quantity of completed activities out of the medium-reach states, but we could not confirm their anomaly status due to limited state administrator input (the interviewee only managed the last year of the grant) and obtaining no documentation.

Three out of seven low-reach states (States 2, 11, and 13) were case anomalies with high quantity and quality activities. State 2 completed 8 out of the 9 activities (88.9%), but these activities did not result in desired outcomes or higher reach. For example, they created a state policy impact report but that did not result in any reported changes in support for either A-CRA implementation or youth SUD treatment in the state.

State 11 described experiencing “extremely high [provider] turnover” which impacted reach despite completing more than half of the activities, as well as concerns about cultural relevance or fit of some A-CRA components:

*“I have heard some concerns that it might not be culturally sensitive, concerns that some of the providers…were like, ‘We can't do some of these. This is not going to float with the community I work with.’ I think that the idea of increasing pro-social recreation is so important, but I'm going to be honest, a lot of times that costs money...Schools are often limited in the resources that they have, the sports and activities. And so a lot of pro-social activities are going to... There's a cost associated with them. And so that can be a challenge when implementing A-CRA.”* (State Administrator Interview, State 11)

And for State 13, their anomaly status was primarily attributable to the fact that their grant supported other EBPs and their grant activities were not specific to implementing A-CRA. Only 2 of the 4 treatment organizations initially involved with the grant opted to deliver A-CRA. A secondary reason was that A-CRA might not have fit with how youth SUD services are structured and supported in this state, which prioritized serving adolescents with SUD in residential programs, rather than outpatient.

### Exemplar activities

From the high-reach cases, we were able to identify high-quality exemplars for each of the nine activities. Table 6 provides a list of exemplar activities that high-reach states established. By contrast, one low-reach state described just having a “train and hope” approach to getting providers certified. High-reach states were more proactive and attentive to mitigating certification challenges or staff turnover. High-reach states also consistently had higher standards and criteria for themselves in terms of completing the grant-specified activities and whether they were satisfied with their reach.

**Table 6:** Exemplar activities from high-reach state cases by EPIS construct

EPIS Construct(s)	Exemplar Activity
Inner Context	Required all participating youth treatment organizations to have at least 1 clinician and 1 clinical supervisor certified, could not be the same person. *(State 8)*
Inner Context	Established quarterly A-CRA learning collaborative that worked together to develop an A-CRA Provider Handbook. *(State 10)*
Inner Context	“Set expectations” before engaging treatment organizations in A-CRA activities (creating documentation and communicating with potential organizations about what A-CRA certification and delivery entails). *(State 10)*
Inner Context	Established effective learning collaborative as a forum for providers to share their experiences with getting certified in A-CRA and treating youth. *(State 12)*
Inner Context	Reduced administrative burden for providers by streamlining associated paperwork and administrative tasks. *(State 14)* *Note: An illustrative quote describing this can be found in the “Impacts of Provider Turnover” row in Table *7
Inner Context	Introduced a “career ladder” to facilitate site sustainability planning/to help mitigate the impacts of provider turnover. *(State 15)*
Inner Context	Even though they did not manage to establish a dedicated in-state trainer as intended, still hired a training coordinator who was responsible for setting up all the trainings with CHS, sending out notifications for training and certification, and making sure providers really understood the commitment level for training and certification prior to initiating the process. *(State 15)*
Inner Context, Bridging Factors	Set explicit requirements for provider and community engagement as part of the RFP they issued for treatment organization sites. Every site was required to do a minimum of 12 outreach activities/year. *(State 14)*
Outer Context	Promoted A-CRA actively and widely in the state through: 1) statewide marketing and brochures to educate the public, 2) promoting A-CRA as a treatment modality in state councils and sub-committees, and 3) purchasing items that providers could use as incentives for youth and families to engage + maintain them in treatment. *(State 3)*
Outer Context	Instituted a new contracting vehicle that supported additional A-CRA specific activities that were not reimbursable through Medicaid (e.g., some of the certification process activities). *(States 10 and 15)*
Outer Context, Bridging Factors	Conducted extensive community engagement by holding local community information sessions with families, juveniles, educators, and other school system staff to talk about youth substance use and A-CRA as an EBP. Also brought in clinicians to talk about delivering A-CRA, and 22–25 year olds in recovery to talk about their experiences during these information sessions. *(State 12)*

### Factors that influenced A-CRA reach

Our analysis of factors that influenced A-CRA reach generated five major findings: 1) provider turnover, 2) state agency staff turnover, 3) grant period, 4) learning collaboratives, and 5) state leadership buy-in and support. Table 7 provides an overview of these findings, along with their relevant EPIS construct, State IDs, influence on reach, and illustrative quotes.

**Table 7:** Overview of five factors that influenced reach with illustrative quotes

Key Finding	EPIS Construct	Relevant State IDs	Reach Barrier/Facilitator	Illustrative Quotes
1. Impacts of Provider Turnover	Inner Context	Low: 1, 2, 7, 11, 13 Medium: 9, 16 High: 10, 14, 15	Barrier	*“I would maybe add just the difficulty of retaining our substance use clinician workforce. That has been heavy pressure on whether or not we can sustain the A-CRA model here because we paid to train clinicians but then they move on or get another job somewhere else.* (State Agency Administrative staff, State 2) “*Requiring the combined GAIN-I* [Global Appraisal of Individual Needs – comprehensive bio-psychosocial assessment] *and GPRA* [Government Performance and Results Act client outcomes measures] *caused a great deal of essentially survey fatigue in clients, and even generated some turnover at one of our partners where a therapist was feeling like we were more interested in collecting data than providing services. So we took that to heart and we switched to the GAIN-Q3* [Global Appraisal of Individual Needs – brief screener]*, and that seemed to move things along at a better pace.”* (Lead Evaluator, State 14)
2. Impacts of Agency Staff Turnover	Inner Context	Low: 1, 2, 13, 19 Medium: 6, 9 High: 5, 10, 12	Barrier	*"Well, I think the change in leadership was not helpful during the grant, as I mentioned before, we had three directors. And so, we were having to sort of pitch A-CRA afresh each time, each short time a new director came into that position."* (State Agency Lead Trainer, State 5)
3. Total # of Grants and Total Length of Grant Period	Bridging Factors	All	*Longer grant period* = Facilitator *Shorter grant period* = Barrier	N/A – derived from grant documentation, not from interviews
4. Learning Collaboratives	Bridging Factors	Low: 11 Medium: None High: 10, 12	Facilitator	*“One of the things that we've struggled with and don't struggle with anymore, but there are other agencies struggling with this, is keeping the workforce. Like turnover. I train them and then they leave…We experienced a lot of that too. And it was because people didn't have a full understanding of what they were signing up for. And so, we really want people to understand this isn't like, I'm going to go to training and then I'm going back to life as usual. You're involved in at least a nine-month process, or sorry, at least a six to nine month process of certification. You're coming to a learning collaborative quarterly. Respondent 2 and I always joke we're like, ‘We're getting married. We're not just dating.’”* (State Agency Administrative staff, State 10) *“So during that period of grant, we didn't necessarily do the A-CRA learning collaborative formalized, but I did provide ongoing implementation support to people that were trained and hoping to get certified. So I know specifically there was a group that I worked with periodically…to get a group of professionals trained in A-CRA. So we did do some, so it wasn't formalized as like collaborative consultation calls, but implementation support was available and offered through me. And sometimes clinicians would connect with me to discuss working towards certification.”* (Training Director for Substance Use Initiatives, State 11)
5. Attentiveness to State Leadership Buy-in and Support for A-CRA	Outer Context	Low: None Medium: 6 High: 5, 12, 14, 15	*Strong leadership buy-in and support* = Facilitator for States 6, 14, 15 *Weak leadership buy-in and support* = neutral, did not negatively impact reach for States 5 and 12	*"Currently, my executive director is a big advocate for serving adolescents in general [and] Obviously, our legislators are very helpful, especially when they inquire annually, they are inquiring about our efforts and how we are reaching the adolescent population, so it helps my state leadership, my supervisors to recognize that, ‘Okay, this is still a concern of legislators and their constituents,’ and so they see that we need to continue to offer the program. Honestly, that's the biggest driving factor.”* (State Agency Administrative staff, State 14)

First, states were most successful in promoting A-CRA reach when they proactively took actions to mitigate provider turnover, which was a consistent inner-contextual implementation barrier. Provider turnover was described as being particularly challenging due to the complicated and typically lengthy process involved with training and becoming A-CRA certified. As previously reported, mean time to first-level certification for clinicians during the state-focused grants was 50.2 weeks, and mean time to full certification was 62.9 weeks [31]. Turnover during or after the certification process, meant that after a new clinician was hired, if the clinician was not already certified in A-CRA, the organization needed to support their time to attend A-CRA training and achieve certification. Provider turnover was reported as a challenge in 5 out of 7 low-reach cases, 2 out of 5 medium-reach cases, and 3 out of 7 high-reach cases (i.e., 53% of the sample). One key distinction, however, is that all 3 of those high-reach cases (States 10, 14, and 15) explicitly described making modifications to mitigate provider turnover. In State 10, they described “socializing” treatment organization sites and providers in advance on the demands of A-CRA certification by establishing learning collaboratives (see Finding #4 below), and changing their requirements to only allow masters-level clinicians to be certified which reportedly made a difference. For State 14, they adjusted the assessment requirements to be briefer and less burdensome on providers and clients (see Table 7).

In State 15, they helped to mitigate the repercussions of provider turnover by introducing a train-the-trainer approach that they called a “career ladder” to support sustainability planning for sites by making sure a trained supervisor was available at every site to help certify and train new providers as turnover happened. They also established an in-state training institute that proved useful in getting new providers trained more quickly. By contrast, none of the medium- or low-reach cases described making these types of modifications when challenged by provider turnover issues.

Second, turnover of staff at the state agency was also a consistently reported outer-contextual implementation barrier. This was experienced by 4 out of 7 low-reach cases, 2 out of 5 medium-reach cases, and 3 out of 7 high-reach cases (i.e., 37% of the sample). For example, one high-reach state (State 5) had three different directors during the grant period. No concrete actions were described or documented for outer context staff turnover. However, we hypothesize that this barrier may end up mattering more for sustainment than it did for reach – for example, in one low-reach state (State 19), the program manager left the state agency just as the grant funding was set to end.

Third, we found that the total number of grants (1 vs. 2) and total length of grant period helped to explain reach patterns as bridging factors. In general, states with longer grant periods (i.e., two grants that were non-overlapping) had better reach than states with shorter grant periods (see Table 3). Out of the 7 low-reach states, 6 had only one grant (mean grant years = 4). Out of the 5 medium-reach states, 3 had only one grant (mean grant years = 4.2). Out of the 7 high-reach states, 3 had only one grant (mean grant years = 4.71).

Fourth, establishing formal learning collaboratives appeared to be related to better reach as a bridging factor that facilitates inter-connections and interactions. Specifically, 2 of the high-reach cases (States 10 and 12) developed learning collaboratives supporting providers throughout the state to learn from each other and share their experiences with getting certified and delivering A-CRA. For example, State 10, held quarterly learning collaboratives that provided a venue to discuss sustainability planning and inter-organizational collaboration. Related to Finding #1, this was described as being effective for addressing their issues with provider turnover. Providers within the learning collaborative developed a state-specific A-CRA provider handbook.

None of the medium-reach states established learning collaboratives. One low-reach state (State 11) mentioned that they conducted informal calls for providers from treatment organizations, but not at the level of a learning collaborative. By their own description, these weren’t formalized and served mainly as ad hoc group consultation calls to seek implementation support from the state, rather than opportunities for providers to share and learn from each other.

Fifth, attention to state leadership A-CRA buy-in and support, an outer-contextual factor, was related to reach. This was documented in 4 out of the 7 high-reach cases, with two states describing positive impacts of strong state leadership support for adolescent SUD treatment and A-CRA (States 14 and 15) and two states describing being able to implement despite lack of state leadership support (States 5 and 12). For State 14, both their state agency’s executive director and state legislators were very focused on serving adolescents in their state. And for State 15, it was their state commissioner of health who actively supported and shared information specifically about A-CRA with providers throughout the state.

In terms of being able to still implement and have high reach despite lack of leadership support, State 12 mentioned that during their grant period, the state governor was not supportive of youth treatment services and did not believe in funding social services in general. Despite this, their own agency leadership were still “really great champions” and “really cared a lot about adolescents and treatment”. For State 5 it was a general lack of support in the state at the executive leadership level – “there is little support in the state for adolescent substance use treatment, it is not a priority.”

Notably, none of the 5 medium-reach states or 7 low-reach states mentioned state leadership buy-in and support in the same ways as those high-reach cases. For one medium-reach state, State 6, there was brief mention of a local champion (a judge) in one region of the state, who advocated for A-CRA and helped to get schools interested. Despite this, they mentioned still having “very low adoption rates.” In sum, high-reach states were attending to state support beyond their agency and were able to do well even despite lack of support at higher levels within their state.

## Discussion

Our comparative case study analysis of a financing strategy to promote EBP implementation to address youth SUD identified nuanced explanations across multiple sources of evidence to help explain reach. We identified five factors that influenced reach outcomes: 1) provider turnover, 2) state agency staff turnover, 3) funding period, 4) learning collaboratives, and 5) state leadership buy-in and support. Our case explanations specifically highlight that quantity *and* quality of state's grant-funded activities influenced reach. By quality, we found that high-reach states had more specific, intentional, and explicit activities and expectations than the low- and medium-reach states.

Provider turnover is an ongoing critical workforce issue in behavioral health services. In SUD treatment research, staff turnover has been found to be as high as 50% in some settings [49], with one longitudinal study finding an annual turnover rate of 33.2% for counselors and 23.4% for clinical supervisors [50]. This is likely to influence not only implementation, but sustainment as well, with one recent scoping review finding that workforce turnover can result in funding instability (substantial financial impacts due to increased need to hire and train new staff), community impacts (loss of continuity of care and trust), and other consequences [51]. Prior studies have found that implementation strategies that support staff and EBP implementation that fit the needs of providers can lead to greater staff retention during Implementation and Sustainment phases [52–54]. We identified several strategies that states used to meaningfully address provider turnover, including adapting certain requirements (i.e., reporting) and criteria (i.e., training eligibility), developing context-specific provider handbooks, or introducing learning collaboratives and “career ladders”. The success of high-reach states, despite turnover, highlights that such high-quality and durable implementation strategies can build more resilient implementation infrastructure that is less dependent on specific individuals.

State agency staff turnover also has impacts on behavioral health service delivery but has been understudied compared to provider turnover. Some research has examined turnover at national government agencies [55, 56], and few have focused on state agencies. One U.S. study found state health agency staff to have the lowest satisfaction (65%), when compared to staff at the national health agency level (67%) and staff at the local and regional health agency level (69%) [57]. They also found intent to leave to be higher for state health agency staff (24%) than for federal agency staff (19%). Further research is needed to understand effective strategies to address state agency staff retention. As mentioned earlier, we hypothesize that this may matter more for sustainment than it did for reach. In future work, we plan to utilize similar methods to examine state sustainment, as EPIS emphasizes the importance of planning for sustainment throughout all phases of implementation and SAMHSA explicitly hoped to support sustainment through these grants [41].

Length of grant funding period is understood to impact EBP implementation and sustainment success, with longer grant periods being critical to maintaining continuity of treatment delivery [4, 32, 58]. Our findings contribute to this by showing that both total number of grants and having longer grant periods, related to higher reach. National government agency officials should consider lengthening grant award periods by allocating funds for the EPIS Preparation phase prior to active implementation, and providing dedicated support for covering the transition between the EPIS Implementation and Sustainment phases (e.g., switching to other funding sources after initial grant funding ends).

Learning collaboratives are increasingly used in behavioral health as a workforce development and implementation strategy [59–61]. Our finding that formal learning collaboratives can serve as a bridging factor facilitating inter-connections and interactions, points to key opportunities to optimize learning collaboratives in future efforts. For example, those employing learning collaboratives should consider using them as a venue to discuss sustainability planning and inter-organizational collaboration, modifications that can mitigate provider turnover and perhaps develop context-specific tools (e.g., provider handbooks).

The impacts of policymaker leadership buy-in and support for behavioral health EBP implementation and sustainability in the face of competing priorities and limited resources has been well documented [62, 63], particularly the vital role that state leaders can play as EBP champions [64] that facilitate EBP delivery. Our findings demonstrate that even when encountering lack of state leadership support for EBP for youth SUD, attentiveness to this EPIS outer context barrier can still make high reach possible.

In summary, this study contributes evidence for effective policy guidance on leveraging grant funding and other financing strategies to optimize EBP reach. Guided by EPIS, this project used mixed-method data and comparative case study methods to understand state efforts to build state infrastructure to support implementation and implement A-CRA – focusing on provider-level reach as the primary outcome. Our study has several strengths that help inform and improve future policy financing strategies for EBP implementation, as well as advance policy implementation research methods. First, the operationalization of reach as a primary implementation outcome in a more nuanced way helps to advance conceptualization of this key implementation outcome in policy contexts [65, 66]. More specifically, we took into account the number of providers adequately trained as well as the context in which they were working (extent of need in their state) to create three levels of reach. Second, our case study design and use of pattern matching maximized the learning possible from the available data. In policy implementation science studies, we are often limited by relatively small samples sizes, lack of randomization, externalities, and dynamics that make simple comparisons unlikely to robustly explain variation in implementation processes and outcomes.

The explanations we generated about financing strategies improves our ability to address common implementation barriers like provider turnover, as not just an issue that inevitably happens, but something that can be meaningfully addressed. National government agencies and other settings seeking to optimize their financing strategies should incorporate monitoring and support for proactive measures to strategically plan for contingencies that help prevent and mitigate barriers such as provider and state agency staff turnover. To attenuate issues of EBP fit (e.g., related to cultural relevance issues, or how youth SUD services are structured and supported in a jurisdiction), financing strategies should also support opportunities to adapt EBP components and their delivery to local contexts while maintaining fidelity; examples could include funding community needs assessments during the Preparation phase, or providing support for an EBP training and certification team representative to meet periodically with learning collaborative members to share implementation experiences from other settings that may address barriers.

Despite the strengths of the current study, there are some limitations to note. First, we focused on provider-level A-CRA reach, rather than client-level reach (youth with SUD receiving A-CRA as a treatment). We did not have access to data on client-level reach. Future research should examine the relationships between levels (e.g., high, medium, low) of provider- *and* client-reach. Second, as noted in the Methods, we had variability in the available data sourced across the 19 state cases, which meant having greater detail and depth available for some states and less information available for others. Also, the research was conducted in the U.S. where federal funding is the primary source for substance use treatment and results may look different in other countries that support EBPs to address youth SUD differently. Third, although we did see a small subset of states sub-contract some grant activities to external organizations, we were not able to derive potential implications about such sub-contracts due to each state sub-contracting to very different entities. Future research with larger samples should examine sub-contracting as an implementation barrier (e.g., assessing when and how sub-contractors increase the likelihood of poor implementation or drift due to the contracted entity not being involved in planning the funded proposal) or an effective implementation strategy (e.g., examining when and how sub-contractors facilitate implementation by leveraging skilled personnel).

## Conclusions

Our mixed-method comparative case study used an innovative approach to conduct policy-focused implementation research to explain variation in implementation across 19 states all supported by the same financing strategy (federal grants to support states to deliver EBP to address youth SUD). These findings have implications for how future grant initiatives and policies can effectively facilitate the financing and implementation of evidence-based treatments.

## Supplementary Information

Additional file 1. StaRI Reporting Checklist.

Additional file 2. Wave 1 Interview Guide.

Additional file 3. Case Summary Template.

Additional file 4. Data Sources for State Characteristics During Grant Period.

## Data Availability

The datasets generated and analyzed during the current study are available from the corresponding author on reasonable request. Depending on the nature of the request, additional data-sharing agreements may be required.

## Abbreviations

A-CRA: Adolescent Community Reinforcement Approach
CHS: Chestnut Health Systems
EBP: Evidence-Based Practice
EHR: Electronic Health Record
EPIS: Exploration, Preparation, Implementation, Sustainment Framework
GAIN: Global Appraisal of Individual Needs
GPRA: Government Performance and Results Act
SAMHSA: Substance Abuse and Mental Health Services Administration
SAT-ED: State Adolescent Treatment and Dissemination Initiative
SUD: Substance Use Disorder
SYT: State Youth Treatment Initiative
SYT-I: State Adolescent and Transitional Youth Treatment and Dissemination Initiative
U.S.: United States
