# Supplementary material for: Clarity and consistency in government-funded implementation strategies associated with greater evidence-based practice reach: a mixed-method comparative case study
Source: Implement Sci. 2025 Dec 22;21:12. doi: 10.1186/s13012-025-01470-3 (PMC12874977; doi:10.1186/s13012-025-01470-3)
Supplement: Supplementary file 2 — Additional file 2. Wave 1 Interview Guide. [file 13012_2025_1470_MOESM2_ESM.docx]

# A-CRA Financing Project: STATE ADMIN INTERVIEW COVER SHEET

RESPONDENT ID(s):

RESPONDENT NAME(s):

STATE ID:

RESPONDANT STATE:

STATE AGENCY NAME:

DATE OF INTERVIEW: ______ / ______ /_______

TIME OF INTERVIEW (INTERVIEWEE TIME): _______ PT/MT/CT/ET

INTERVIEWER:

PHONE NUMBER FOR INTERVIEW:

E-MAIL:

A-CRA GRANTS RECEIVED:

SAT-ED START DATE: ______ / ______ /_______ END DATE: ______ / ______ /_______

SYT START DATE: ______ / ______ /_______ END DATE: ______ / ______ /_______

SYT-I START DATE: ______ / ______ /_______ END DATE: ______ / ______ /_______

STR START DATE: ______ / ______ /_______ END DATE: ______ / ______ /_______

YT-I START DATE: ______ / ______ /_______ END DATE: ______ / ______ /_______

other START DATE: ______ / ______ /_______ END DATE: ______ / ______ /_______

(if other, describe: ___________________ )

OTHER NAMES FOR THE SAMHSA/CSAT-FUNDED A-CRA IMPLEMENTATION PROJECT IN THIS STATE:

RELEVANT CASE NOTES:

***STATE ADMIN INTERVIEW VERBAL CONSENT***

(read prior to beginning interview/recording)

Hi [STATE ADMIN NAME],

This is [INTERVIEWER NAME] calling from RAND to speak with you about [STATE AGENCY NAME]’s experiences with implementing the Adolescent Community Reinforcement Approach (A-CRA) treatment model in [STATE]. Is this still a good time to complete your interview? To ensure confidentiality, make sure you are in a private, secure location for completing the interview.

>IF NO, reschedule.

>IF YES, great.

We would like a representative from [STATE AGENCY NAME] to answer these questions. Ideally, it should be someone knowledgeable about your A-CRA implementation efforts. Are you the right person at your agency to participate in this opportunity?

>IF NO: May I get the contact information of the best person to speak with about the state’s youth/adolescent substance use treatment programs (especially A-CRA implementation)? [RECORD CONTACT INFORMATION TO FOLLOW-UP]

>IF YES, thanks for confirming.

Before we begin, let me assure you that your responses to these questions will be held in strict confidence. In collaboration with Chestnut Health Systems, we are requesting interviews with state agency representatives in nearly 20 states that received funding from the SAMHSA Centers for Substance Abuse Treatment (CSAT) to deliver A-CRA. We will use information from the interviews to understand how different CSAT funding models influence the implementation and sustainability of A-CRA. We will also examine information provided by treatment organizations that implemented A-CRA as part of CSAT grants like yours through surveys and interviews. We will not attribute comments to specific individuals or programs in any of our reports or publications. Your responses will not be shared with your agency or with SAMHSA.

Today’s interview will last about 60 minutes. You will receive a $25 Amazon e-gift card as a thank you for your participation.

Your participation in this discussion is entirely voluntary. We would like to have your responses to all of the questions. However, if you’re uncomfortable with any question we can skip it, and you can stop the interview at any time. There are no right or wrong answers – we are interested in your perspectives and experiences. After the interview, we may follow up with you about sharing documentation related to your CSAT grant that would be useful for us to review. It will always be your decision whether to share. Finally, we would like to audio-record the interview to ensure that we capture everything that is said. We will destroy the recording once we confirm we have captured everything in our de-identified notes and transcripts. However, you can still participate in the interview even if you do not give permission to audio-record.

If you have any questions or want to discuss the project further at any time, you may always contact us at [PHONE] or at [EMAIL]. Furthermore, if you have questions about your rights as a research participant or need to report a research-related injury or concern, you can contact RAND's Human Subjects Protection Committee toll-free at (866) 697-5620 or email [hspcinfo@​rand.org](mailto:hspcinfo@​rand.org)​. If you contact the Committee, please reference Study #2020-N0887.

- **Do you have any questions?**
- **Are you willing to take part in this discussion?**

>IF NO: That is not a problem, thank you for your time.

>IF YES, great.

- **Is it ok with you if we audiotape this discussion?**

>IF NO: That is not a problem. I can take notes while we’re talking so I don’t miss anything important, though that means we might proceed through the interview more slowly than usual. I could also arrange for a colleague to take notes during the interview. [RESCHEDULE IF NEEDED]

>IF YES, perfect, let’s get started.

**Interview Protocol – STATE BEHAVIORAL HEALTH AGENCY ADMINISTRATOR**

1. Tell me a little bit about the state agency where you work. What types of services does your agency oversee or administer? (e.g., age groups, problem types, etc.)
2. What is your primary professional role(s) at your agency? (What are you responsible for overseeing?)
3. At the state agency, have you worked on any SAMHSA/CSAT-funded projects to disseminate A-CRA? Yes or No?

🞏 No **– *SKIP TO QUESTION 4***🞏 Yes – ***CONTINUE***

3A. Between what dates did you work on the project(s)? Your best guess is fine.

1. What do you think of A-CRA as a treatment for youth – does it meet the needs of the populations you serve? How so or why not? Of note, throughout this interview, by “youth” we typically mean ages 12 through 17 (or through age 24 if A-CRA training in your state included young adults).
2. What do think of A-CRA as a treatment for outpatient youth substance use treatment services – does it fit well into treatment organizations’ services and capabilities? How so or why not?
3. How has your state agency selected treatment organizations that you worked with to support A-CRA implementation?
4. Next, I’m going to list seven activities that your state agency might have engaged in its efforts to help provider organizations implement A-CRA. Please let me know whether your state agency engaged in each of these activities, and if so, any details you know about what was done and when.

7A. Providing training for organizations to implement A-CRA?

🞏 No
🞏 Yes - during the SAMHSA CSAT grant period

🞏 Yes - currently engaging in this activity

🞏 Yes - planning to continue over the coming year

🞏 Don't Know

IF YES, please describe *[PROBE AS NEEDED: What have those activities looked like in your state? Who and what have been involved? How has the activity changed since the grant funding period, if at all?]:*

7B: Directly funding organizations to implement A-CRA?

🞏 No
🞏 Yes - during the SAMHSA CSAT grant period

🞏 Yes - currently engaging in this activity

🞏 Yes - planning to continue over the coming year
🞏 Don't Know

IF YES, please describe *[PROBE AS NEEDED: What have those activities looked like in your state? Who and what have been involved? How has the activity changed since the grant funding period, if at all?]:*

7C: Developing state policies that promote use of A-CRA?

🞏 No
🞏 Yes - during the SAMHSA CSAT grant period

🞏 Yes - currently engaging in this activity

🞏 Yes - planning to continue over the coming year
🞏 Don't Know

IF YES, please describe *[PROBE AS NEEDED: What have those activities looked like in your state? Who and what have been involved? How has the activity changed since the grant funding period, if at all?]:*

7D: Supporting organizations’ sustainability planning for use of A-CRA?

🞏 No
🞏 Yes - during the SAMHSA CSAT grant period

🞏 Yes - currently engaging in this activity

🞏 Yes - planning to continue over the coming year
🞏 Don't Know

IF YES, please describe *[PROBE AS NEEDED: What have those activities looked like in your state? Who and what have been involved? How has the activity changed since the grant funding period, if at all?]:*

7E: Supporting organizations’ efforts to promote youth/family engagement?

🞏 No
🞏 Yes - during the SAMHSA CSAT grant period

🞏 Yes - currently engaging in this activity

🞏 Yes - planning to continue over the coming year
🞏 Don't Know

IF YES, please describe *[PROBE AS NEEDED: What have those activities looked like in your state? Who and what have been involved? How has the activity changed since the grant funding period, if at all?]:*

7F: Promoting organizations’ capacity for inter-organizational coordination?

🞏 No
🞏 Yes - during the SAMHSA CSAT grant period

🞏 Yes - currently engaging in this activity

🞏 Yes - planning to continue over the coming year
🞏 Don't Know

IF YES, please describe *[PROBE AS NEEDED: What have those activities looked like in your state? Who and what have been involved? How has the activity changed since the grant funding period, if at all?]:*

7G: Promoting organizations’ improving their computer systems or electronic health records?

🞏 No
🞏 Yes - during the SAMHSA CSAT grant period

🞏 Yes - currently engaging in this activity

🞏 Yes - planning to continue over the coming year
🞏 Don't Know

IF YES, please describe *[PROBE AS NEEDED: What have those activities looked like in your state? Who and what have been involved? How has the activity changed since the grant funding period, if at all?]:*

7H: Are there other activities or resources your state agency has provided to support A-CRA implementation?

🞏 No
🞏 Yes - during the SAMHSA CSAT grant period

🞏 Yes - currently engaging in this activity

🞏 Yes - planning to continue over the coming year
🞏 Don't Know

IF YES, please describe *[PROBE AS NEEDED: What have those activities looked like in your state? Who and what have been involved? How has the activity changed since the grant funding period, if at all?]:*

1. What implementation activities have you personally been involved in as part of state efforts to implement A-CRA?

8A. Providing training for organizations to implement A-CRA?

🞏 No
🞏 Yes

8B. Directly funding organizations to implement A-CRA?

🞏 No
🞏 Yes

8C. Developing state policies that promote use of A-CRA?

🞏 No
🞏 Yes

8D. Supporting organizations’ sustainability planning for use of A-CRA?

🞏 No
🞏 Yes

8E. Supporting organizations’ efforts to promote youth/family engagement?

🞏 No
🞏 Yes

8F. Promoting organizations’ capacity for inter-organizational coordination?

🞏 No
🞏 Yes

8G. Promoting organizations’ improving their computer systems or electronic health records?

🞏 No
🞏 Yes

8H. Other activities or resources your state agency currently provides to support A-CRA implementation?
🞏 No
🞏 Yes

1. How has your state agency obtained funding for continuing the A-CRA implementation activities or resources that you started during the SAMHSA CSAT grant? These are the activities and resources discussed in the previous series of questions – beyond paying for services. I’m going to read five options, please let me know which ones your agency has used.

9A. Internal funding from agency's budget?

🞏 No
🞏 Yes
🞏 Don't Know

IF YES, please describe:

9B. State budget appropriations?

🞏 No
🞏 Yes
🞏 Don't Know

IF YES, please describe:

9C. Block grant funds?
🞏 No
🞏 Yes
🞏 Don't Know

IF YES, please describe:

9D. Federal grants (like SAMHSA CSAT)?

🞏 No
🞏 Yes
🞏 Don't Know

IF YES, please describe:

9E. Payments from a special funding source (for example, a dedicated tax or bond, settlement fund, etc.)?

🞏 No
🞏 Yes
🞏 Don't Know

IF YES, please describe:

9F. Other sources of funding?

🞏 No
🞏 Yes
🞏 Don't Know

IF YES, please describe:

1. Currently, what funding options does your state agency offer provider organizations to support youth substance use treatment delivery in general?
2. What funding options, if any, does your agency currently offer to support provider organizations that deliver A-CRA specifically? By this, I mean funding for A-CRA activities or services begun during the SAMHSA CSAT grant.
3. The next few questions ask about how successful your state effort has been at implementing A-CRA. Please answer to the best of your knowledge, in the organizations/settings you would know about. I will ask you about success in three areas.

12A. How successful has your state effort been in training clinicians in A-CRA at provider organizations in your state?

12B. How successful has your state effort been in getting clinicians certified in A-CRA?

12C. How successful has your state effort been in youth receiving A-CRA in your state?

1. Tell me about any ways in which your state agency has collaborated with other entities to support A-CRA.

13A. Which of these collaborations is your agency continuing, if any? Why or why not?

1. Tell me about any efforts or planning at your state agency to obtain resources for continuing A-CRA beyond your state’s grant period. By resources, we mean things needed to support A-CRA such as money, staff, supervision, training, and A-CRA manuals.

14A. Which of these efforts or planning is your agency continuing, if any? Why or why not?

1. Can you please describe any state or federal policies that have supported A-CRA delivery? These could include funding and billing rules that support A-CRA delivery. Include policies at your state agency and other agencies.

15A. Which of these policies are currently supporting A-CRA delivery, if any?

1. What about state or federal policies that interfere with A-CRA delivery? (Including at your agency and other agencies)

16A. Which of these policies are currently impacting A-CRA delivery, if any?

1. Can you tell me about any pressure your state agency has experienced to continue delivering A-CRA or to discontinue its use?
2. Would you say provider organizations in your state are generally supportive or reluctant to use A-CRA? Can you give me an example?
3. Next, I would like to know what factors helped efforts to implement and sustain A-CRA in your state. Please let me know if these factors were important in your state, and if yes, how so.

19A. Would you say characteristics of A-CRA helped efforts to implement and sustain A-CRA in your organization? Yes or No?

🞏 No
🞏 Yes

IF YES, please describe: When was that factor most impactful? Was it during the CSAT grant funding period, after the CSAT funding ended, or throughout?

19B. Would you say characteristics of key individuals helped efforts to implement and sustain A-CRA in your organization? Yes or No?

🞏 No
🞏 Yes

IF YES, please describe: When was that factor most impactful? Was it during the CSAT grant funding period, after the CSAT funding ended, or throughout?

19C. Would you say characteristics of treatment organizations helped efforts to implement and sustain A-CRA in your organization? Yes or No?

🞏 No
🞏 Yes

IF YES, please describe: When was that factor most impactful? Was it during the CSAT grant funding period, after the CSAT funding ended, or throughout?

19D: Would you say helpful factors within your state agency helped efforts to implement and sustain A-CRA in your organization? Yes or No?

🞏 No
🞏 Yes

IF YES, please describe: When was that factor most impactful? Was it during the CSAT grant funding period, after the CSAT funding ended, or throughout?

19E: Would you say helpful factors outside your state agency helped efforts to implement and sustain A-CRA in your organization? Yes or No?

🞏 No
🞏 Yes

IF YES, please describe: When was that factor most impactful? Was it during the CSAT grant funding period, after the CSAT funding ended, or throughout?

19F: Would you say partnerships with other agencies or organizations helped efforts to implement and sustain A-CRA in your organization? Yes or No?

🞏 No
🞏 Yes

IF YES, please describe: When was that factor most impactful? Was it during the CSAT grant funding period, after the CSAT funding ended, or throughout?

19G: Were there other helpful factors for implementing and sustaining A-CRA?

🞏 No
🞏 Yes

IF YES, please describe: When was that factor most impactful? Was it during the CSAT grant funding period, after the CSAT funding ended, or throughout?

1. Now, I’m going ask how the same six categories of factors may have included barriers that hindered efforts to implement and sustain A-CRA in your state. Please let me know if these factors were important in your state, and if yes, how so.

20A. Would you say characteristics of A-CRA hindered efforts to implement and sustain A-CRA in your organization? Yes or No?

🞏 No
🞏 Yes

IF YES, please describe: When was that factor most impactful? Was it during the CSAT grant funding period, after the CSAT funding ended, or throughout?

20B. Would you say challenges with key individuals hindered efforts to implement and sustain A-CRA in your organization? Yes or No?

🞏 No
🞏 Yes

IF YES, please describe: When was that factor most impactful? Was it during the CSAT grant funding period, after the CSAT funding ended, or throughout?

20C. Would you say challenges with treatment organizations hindered efforts to implement and sustain A-CRA in your organization? Yes or No?

🞏 No
🞏 Yes

IF YES, please describe: When was that factor most impactful? Was it during the CSAT grant funding period, after the CSAT funding ended, or throughout?

20D: Would you say challenges within your state agency hindered efforts to implement and sustain A-CRA in your organization? Yes or No?

🞏 No
🞏 Yes

IF YES, please describe: When was that factor most impactful? Was it during the CSAT grant funding period, after the CSAT funding ended, or throughout?

20E: Would you say challenges outside your state agency hindered efforts to implement and sustain A-CRA in your organization? Yes or No?

🞏 No
🞏 Yes

IF YES, please describe: When was that factor most impactful? Was it during the CSAT grant funding period, after the CSAT funding ended, or throughout?

20F: Would you say issues in partnerships with other agencies or organizations hindered efforts to implement and sustain A-CRA in your organization? Yes or No?

🞏 No
🞏 Yes

IF YES, please describe: When was that factor most impactful? Was it during the CSAT grant funding period, after the CSAT funding ended, or throughout?

20G. Were there other barriers to implementing and sustaining A-CRA?

🞏 No
🞏 Yes

IF YES, please describe: When was that factor most impactful? Was it during the CSAT grant funding period, after the CSAT funding ended, or throughout?

**Next, I’m going to ask you a few questions about the COVID-19 pandemic.**

1. How has the COVID-19 pandemic affected substance use treatment services in your state?
2. When the pandemic began in March 2020, were any treatment organizations delivering A-CRA in your state?

🞏 No – ***IF NO, SKIP TO QUESTION 25***
🞏 Yes – ***CONTINUE***

🞏 Don’t know – ***SKIP TO QUESTION 25***

1. What have been the most significant changes affecting A-CRA programs in your state due to COVID-19? [PROBE AS NEEDED]
2. Have there been changes made in response to COVID-19 that have been beneficial, and will those changes continue beyond the pandemic? [PROBE AS NEEDED]

**OK, we are almost done. Next, I want to ask you about your general impressions regarding the SAMHSA CSAT-funded A-CRA project and then have a few questions about you.**

1. If you had a chance to participate in a SAMHSA CSAT project again, would you consider it?

🞏 No
🞏 Yes

1. Why/why not?
2. Is there anything you would change about the SAMHSA CSAT project in order to improve the sustainment of A-CRA in your state?
3. How many years of experience do you have in the administration of substance use treatment services?
4. How many years of experience do you have in your current position and similar positions/levels? This would include experience at other agencies, if relevant.
5. Do you have any experience as a provider of substance use treatment services?

🞏 No – ***IF NO, SKIP TO QUESTION 32***

🞏 Yes – ***CONTINUE***

1. How many years of experience do you have as a provider?
2. Is there anything else you would like to share related to the topic of sustaining A-CRA in your state?
3. Are there written reports or other documentation that you could share with us that might give us insight into your state’s A-CRA initiative? This could include anything that helps us understand the supports provided by your state agency and/or the services provided by treatment organizations that implemented A-CRA. Documents from the grant period are useful, as are documents from the time period since the grant ended. Even redacted or generic/incomplete documents could be very useful to us. I will send you an email after this call with a list, and you can let us know if they’d be available to share and any relevant considerations.

[IF TIME] Here is a list of documents that state agencies might be able to share. Would you be able to share any of the following? If so, can you describe the documents and any next steps for sharing them?

- CSAT grant progress reports and/or applications?
- Data or information related to CSAT grant progress, such as evaluation reports?
- Contracts with A-CRA treatment organizations, from during the CSAT grant?
- Contracts with A-CRA treatment organizations, from after the CSAT grant?
- Planning tools, e.g., “financial maps” of A-CRA funding, from during the CSAT grant?
- Planning tools, e.g., “financial maps” of A-CRA funding, from after the CSAT grant?
- Information disseminated about your state A-CRA initiative for a broader audience? For example:
- COVID-19 response plans relevant for A-CRA?
- Other written reports or documentation that might give us insight into your state’s A-CRA initiative?

[TURN OFF RECORDER]

That's all the interview questions I have. Thank you for providing this information. We will be sending your $25 Amazon e-gift card by e-mail within the next few days. [IF STATE ADMINISTRATOR INTERVIEW OR GROUP INTERVIEW, FIGURE OUT WHO THE GC SHOULD GO TO (ONLY ONE PER STATE). IT IS POSSIBLE THEY CANNOT ACCEPT THE GC.]

[IF PARTICIPANT WILL BE INTERVIEWED AGAIN, complete the tracking module beginning on the final page]
