# Supplementary material for: Clarity and consistency in government-funded implementation strategies associated with greater evidence-based practice reach: a mixed-method comparative case study
Source: Implement Sci. 2025 Dec 22;21:12. doi: 10.1186/s13012-025-01470-3 (PMC12874977; doi:10.1186/s13012-025-01470-3)
Supplement: Supplementary file 3 — Additional file 3. Case Summary Template. [file 13012_2025_1470_MOESM3_ESM.docx]

**A-CRA Reach Case Summary Template**

***Analysis Information***

| Completed by: | Date start: | Date finish: | Reviewed by: | Date reviewed: | Date finalized: |
| --- | --- | --- | --- | --- | --- |
|  |  |  |  |  |  |

***State Summary Information***

***Youth Substance Use Disorder (SUD) Prevalence***

- ***Grant Start Year:***
- ***Grant End Year:***

***State Substance Use Budget***


***Medicaid Expansion State? (Yes/No; If Yes, when did expansion occur (MM/YY)***


***State Reach***

| ***Total number of people in the state with any certification:*** |  |
| --- | --- |
| ***Total number of people sent for training during grant period:*** |  |
| ***Percentage of people sent who got any certification:*** |  |
|  | |
| ***Number of agencies with at least one person with any certification:*** |  |
| ***Number of agencies in the state that sent people for training:*** |  |
| ***Percentage of agencies with at least one person certified:*** |  |
|  | |
| ***SUD prevalence for ages 12-17 (in thousands):*** |  |
| ***Number of youth w/ SUD that would need to be served by each person in the state with any certification:*** |  |
|  | |
| ***Final Reach Level (Low/Medium/High):*** |  |

***State Agency Summary Information***

| State_Agency_ID | State | Type of State-Focused Grant(s) | Start date | End date |
| --- | --- | --- | --- | --- |
|  |  |  |  |  |
|  |  |  |  |  |

(*add rows as necessary*)

| Brief description of state agency (summarize what services are available at this site, the types of clients served, and the age range of youth A-CRA is used with): |
| --- |
|  |

| Respondent_ID | Did the respondent work in agency during grant period (Y/N) | List of Interviews | | |
| --- | --- | --- | --- | --- |
|  |  | Wave 1 date | Wave 2 date | Wave 3 date |
|  |  |  |  |  |

(*add rows as necessary*)

***History Chart***

| 2012 | 2013 | 2014 | 2015 | 2016 |
| --- | --- | --- | --- | --- |
|  |  |  |  |  |
| 2017 | 2018 | 2019 | 2020 | 2021 |
|  |  |  |  |  |
| 2022 | 2023 | 2024 | 2025 |  |
|  |  |  |  |  |

*Instructions*:

- “G+” = year any continuous funding period(s) started for A-CRA implementation
- “G- “= year any continuous funding period(s) ended for A-CRA implementation

(*Note: “continuous funding” may include multiple grants back-to-back or overlapping*)

- Show interviews with Respondent_ID and wave (e.g., “1111 W1”)
- Insert state document(s) file name by year released/published

***State Documents (# available)***

- *Insert File Name*
- *Insert File Name*

**Research Question (RQ) 1: What did the state do with their grant? Is there variation in infrastructure development among the grantee states?**

| ***Element*** | ***Initial Summary of Evidence*** | ***Data Source(s)*** |
| --- | --- | --- |
| What infrastructure did they establish? *(Use Checkboxes)* | Full-time staff position  Training requirements  Policy and regulation development  Site sustainability planning  State-specific strategic financial planning (financial map)  Family / youth engagement structures  Inter-organization collaboration  Computer Systems / Electronic Health Record (EHR) improvement  Other activities |  |
| Who was involved?  Did they hire at least 1 full-time staff position dedicated to managing this program?  Was there turnover during the grant period? |  |  |
| Provide A-CRA training to designated treatment organizations?  *(i.e., Did they create a state-wide multi-year workforce training implementation plan to provide training? Did they provide cross-agency training? Did they provide continuing education events?)* |  |  |
| Provide funding to treatment organizations to implement A-CRA? |  |  |
| Developed state policies to promote the use of A-CRA? |  |  |
| State-specific strategic financial planning? |  |  |
| Supported organizations’ sustainability planning for the use of A-CRA? |  |  |
| Supported organizations’ effort to promote youth/family engagement?  *(i.e., did they include youth and family in their coordination efforts? – see next item)* |  |  |
| Promoted organizations’ capacity for inter-organizational coordination?  *(i.e., Did they link + coordinate with other child-serving systems through establish a council or adding to an existing one?)* |  |  |
| Promoted organizations’ computer systems or electronic health record improvements? |  |  |
| Were there other activities that the state agency provided to support A-CRA implementation?  *(i.e., Preparing faculty in college and educational settings; Develop standards for licensure, certification, accreditation of programs)* |  |  |
| Has the state obtained funding **during the grant-funded period** to continue A-CRA implementation activities or resources that were started during grant period?  *These could be activities/resources beyond paying for services.* |  |  |

**Research Question (RQ) 2: Which factors, and at/across what levels, influenced A-CRA reach within the state?**

| ***Element*** | ***Initial Summary of Evidence*** | ***Data Source(s)*** |
| --- | --- | --- |
| Matching Status - (Match vs. Mismatch / Anomaly Identification)  To what extent was there matching between # and type of activities in RQ1 and reach rate obtained?   - *Matching* = if # or quality of activities matches the extent of reach (both low/medium/high) - *Mismatch/Anomaly* = if unexplained low reach (high # or quality of activities low reach) OR unexplained high reach (low # or quality of activities and high reach |  |  |
| Which factors seemed to have greater influence on reach? | Innovation |  |
|  | Inner Setting |  |
|  | Outer Setting |  |
|  | Bridging Factors |  |
|  | Interconnections |  |
| Are there other differences (as reported by state administrators, in obtained documentation and/or by treatment sites) in the state that help explain variation in reach rates? |  |  |
| To what extent are they satisfied with their A-CRA reach? |  |  |
| To what extent would (if known non-sustainer or unknown sustainment status) or will (if known sustainer) A-CRA reach be approached differently in the future? |  |  |
| **Other implementation outcomes of interest:**   \| Grant Fidelity \| \| --- \| \| Adoption *(did organizations apply or did the state pick them?)* \| \| Fit *(Acceptability, Feasibility, Appropriateness)* \| | Fidelity |  |
|  | Adoption |  |
|  | Fit |  |
