# Supplementary material for: Clarity and consistency in government-funded implementation strategies associated with greater evidence-based practice reach: a mixed-method comparative case study
Source: Implement Sci. 2025 Dec 22;21:12. doi: 10.1186/s13012-025-01470-3 (PMC12874977; doi:10.1186/s13012-025-01470-3)
Supplement: Supplementary file 4 — Additional file 4. Data Sources for State Characteristics During Grant Period. [file 13012_2025_1470_MOESM4_ESM.docx]

**Data sources for state characteristics during grant period**

| **State Characteristic** | **Data Source** |
| --- | --- |
| State Population Size | U.S. Census Bureau: 2010-2019 <https://www.census.gov/data/tables/time-series/demo/popest/2010s-state-total.html> |
| Youth SUD Prevalence | SAMHSA National Survey on Drug Use and Health (NSDUH) State Reports: <https://www.samhsa.gov/data/nsduh/state-reports> |
| State Substance Use Budget | Pew: <https://www.pewtrusts.org/~/media/assets/2015/03/substanceusedisordersandtheroleofthestates.pdf> |
| Medicaid Expansion | Kaiser Family Foundation: <https://www.kff.org/medicaid/issue-brief/status-of-state-medicaid-expansion-decisions-interactive-map/> |
